# Overview of RAW264.7 for osteoclastogensis study: Phenotype and stimuli

**DOI:** 10.1111/jcmm.14277

**Published:** 2019-03-20

**Authors:** Lingbo Kong, Wanli Smith, Dingjun Hao

**Affiliations:** ^1^ Department of Spine, School of Medicine Honghui‐hospital, Xi’an Jiaotong University Xi’an China; ^2^ Department of Neuroscience Johns Hopkins University Baltimore Maryland

**Keywords:** bone marrow macrophage, LPS, osteoclast, phenotype, RANKL, RAW264.7, TNF‐a

## Abstract

Bone homeostasis is preserved by the balance of maintaining between the activity of osteogenesis and osteoclastogenesis. However, investigations for the osteoclastogenesis were hampered by considerable difficulties associated with isolating and culturing osteoclast in vivo. As the alternative, stimuli‐induced osteoclasts formation from RAW264.7 cells (RAW‐OCs) have gain its importance for extensively osteoclastogenic study of bone diseases, such as rheumatoid arthritis, osteoporosis, osteolysis and periodontitis. However, considering the RAW‐OCs have not yet been well‐characterized and RAW264.7 cells are polymorphic because of a diverse phenotype of the individual cells comprising this cell linage, and different fate associated with various stimuli contributions. Thus, in present study, we provide an overview for current knowledge of the phenotype of RAW264.7 cells, as well as the current understanding of the complicated interactions between various stimuli and RAW‐OCs in the light of the recent progress.

## 1 INTRODUCTION

The integrity of bone and skeleton tissue is preserved by the balance maintaining between the activity of the osteoblasts conducted bone forming and osteoclasts conducted bone resorbing, which ensures no net change in bone mass.^1^, ^2^ However, the balance was constantly cycled for remodelling bone tissue with the starting from the osteoclasts formation (osteoclastogenesis),^3^ and investigations for the osteoclastogenesis were hampered by considerable difficulties associated with isolating and culturing osteoclast in vitro.^4^ To solve the issues of osteoclast differentiation in vitro, initial studies try to differentiate osteoclasts through co‐culturing various cell types, such as splenocyte precursor cells, primary monocytes/macrophages with primary osteoblasts or stromal cell linages,^5^, ^6^ which subsequently followed the collagenase treatment, to allow releasing of the osteoblast/stromal cell component, finally leading to the formation of osteoclastic cells.^7^ However, except few cell linages could be used for decipher the mechanisms involved in osteogenesis and bone homeostasis, the most cell linages finally be proved as an invaluable research cellular tool for study osteoclastogenesis.^4^

Osteoclasts arise in the bone marrow from the fusion of haematopoietic cells of a monocyte/macrophage lineage after stimulation by macrophage colony‐stimulating factor (M‐CSF)^8^ and receptor activator of nuclear factor‐κB ligand (RANKL).^9^ RANKL acts directly on osteoclast precursors, via the receptor RANK, to induce differentiation of precursors to multinuclear bone resorbing cells.^10^ As the consequence of the discoveries and extensively exploring for M‐CSF and RANKL, study for osteoclasts in the fields of cellular developing, functional activity and biological molecular mechanism stepped into new era.^11^, ^12^, ^13^ Primary osteoclast precursor cells, which including bone marrow macrophage (BMMs),^14^ splenocytes^5^ and peripheral blood monocytes,^15^, ^16^ can now be induced to osteoclastic differentiation in vitro by culturing in the presence of recombinant M‐CSF and RANKL.^17^, ^18^ However, the use of these primary cells raised difficulties, which associated with these cell linage characters, such limits including the availability and variation in response patterns among different cellular study preparations.^19^, ^20^ Moreover, as a genetic virtually untransfectable cell linage, primary cell‐derived osteoclasts and their precursors are poorly suited for genetic manipulation and promoter studies.^20^

In addition to primary cells, the RAW264.7 cells are monocyte/macrophage like cell linage, originating from *Abelson leukemia* virus transformed cell linage derived from BALB/c mice.^21^ Initially, RAW264.7 cells have been described as an appropriate model of macrophages, as the cell are capable for performing pinocytosis and phagocytosis.^21^, ^22^ Later, further studies proved RAW264.7 cells could respond to stimuli in vitro and subsequently generate multinucleated cells with the hallmark characteristics expected for fully differentiated osteoclasts (RAW‐OCs).^23^ RAW‐OCs have been extensively employed in studies of osteoclastogenesis for more than 20 years. In fact, as the pioneers of this field Collin‐Osdoby and Osdoby pointed out, in parallel or as a prelude to primary cells induced osteoclasts, such as bone marrow macrophages derived osteoclast (BMM‐OCs), RAW‐OCs presenting many advantages of osteoclastogenic cellular model system over the BMM‐OC populations, mainly including (a) easy culture and passage; (b) widespread availability of this cell line to most researchers; (c) homogeneous nature of the osteoclast precursor populations (devoid of osteoblast, stromal, lymphocytes, or other cell types; (d) close correlation in characteristics, gene expression, signalling and developmental or functional processes among the RAW‐OCs, primary precursor cell linage derived osteoclasts and isolated in vivo formed osteoclasts.^4^ Therefore, RAW‐OCs can be used for studying osteoclastogenesis through different methods for various study purposes, including: biochemical, immunological, physiological, molecular and functional assays according to various study procedures.

RAW264.7 cell linage is well‐characterized with regard to macrophage‐mediated immune, metabolic and phagocytic functions^21^ and is increasingly used and accepted as a cellular model of osteoclastogenic study;^24^, ^25^, ^26^ however, with the vastly usage of RAW‐OCs for understanding the osteocalstogenesis in the past two decades, there raised considerable requirements for the extensively understanding the RAW‐OCs and associated cellular biological mechanisms.^27^, ^28^ On the other hand, document studies reported that during the osteoclastic induction, various stimuli might lead different cellular fates during the RAW‐OC induction.^29^, ^30^, ^31^ Considering RAW264.7 cell‐derived osteoclasts have not yet been well‐characterized, and RAW264.7 cells are polymorphic with respect to the phenotype of the individual cells comprising this cell line and different results associated with various stimuli contributions.^32^, ^33^, ^34^, ^35^ Therefore, in our present review, we will summarize the current knowledge of the phenotype of RAW264.7 cells and the current understanding of complicated interactions between various stimuli and RAW‐OCs in the light of the recent progress in this field.

## 2 PHENOTYPE STUDY OF RAW264.7 AND ITS ROLE ON THE REGULATION OF RAW‐OCS

Macrophages are very sensitive to environmental conditions, specifically, in response to different stimuli signals, macrophages (M0 or Mφ)^36^ can display different functional phenotypes including classically activated (M1 or pro‐inflammatory) and alternatively activated (M2 or anti‐inflammatory) phenotypes.^37^, ^38^, ^39^ Established RAW264.7 cells as an immortalized monocyte/macrophage cell lineage,^40^, ^41^ its phenotype might change with the passages and micro‐environments of the cell;^32^, ^42^ therefore, there raise a cautious for interpretation of data obtained from experiments conducted only on the established cell lines, which also including RAW‐OCs. On the other hand, their stability between various laboratories and passages is questionable.^43^ American Type Culture Collection, the main supplier of the cell lines, recommends the passage using are controlled above for passage No. 18, since study showing the induction efficacy might decrease with passage and consequently phenotype changing.^4^, ^19^, ^32^ Otherwise, previous study showed the elder RAW264.7 cells could change its morphology and decrease the production of proteins, which lead RAW264.7 cells resistant for differentiation and transduction.^44^ However, there still lack evidence for rigid classification criteria of cell lines between various passages and it seems to be cell‐linage dependent. Therefore, combined with previous excellent and novel studies in these fields,^4^, ^32^ in our experiences, it might be a reason for different RAW‐OC induction efficacies among various study groups. Therefore, confirmation of RAW264.7 stability through phenotype study among is important for the proper further data interpretation.^32^

Collin‐Osdoby et al^4^ have firstly reported that the recommendation of RAW264.7 cell line up to passage No. 18, due to decreasing efficacy for RAW‐OCs production. To clarify the phenotype stability of RAW264.7 macrophages, Taciak et al^32^ performed systematic analyses of RAW264.7 gene expression with a panel of 28 gene, which contained with macrophage metabolisms. Their study results manifested that through the passages the expression of genes in RAW264.7 from passage No. 5 to No. 50 should be classified into three expression sub‐panels, which include (a) increasing expression; (b) stable expression; and (c) fluctuating expression.^32^ Specifically, the genes from subpanel‐1 are involved in macrophage functions (including: CD11b, CD14, Ireb‐2, TfR (transferrin receptor), CD36, iNOS, CD11c, VEGFR2 (vascular endothelial growth factor receptor 2), TRAP (tartrate‐resistant acidic phosphatase), TIM‐2 (T‐cell immunoglobulin and mucin domain 2) and HIF‐2α (hypoxia inducible factor‐2α)), which demonstrated a significantly stable from passage No. 5 to No. 50.^32^ However, comparing to RAW264.7, the bone marrow macrophage‐M0 (BMM‐Mφ) has been reported markedly express CD169.^45^, ^46^, ^47^ Otherwise, the CD11c and iNOS in subpanel‐1 are demonstrated as highly expressed in pro‐inflammatory macrophage stage (M1).^48^

**Figure 1:**
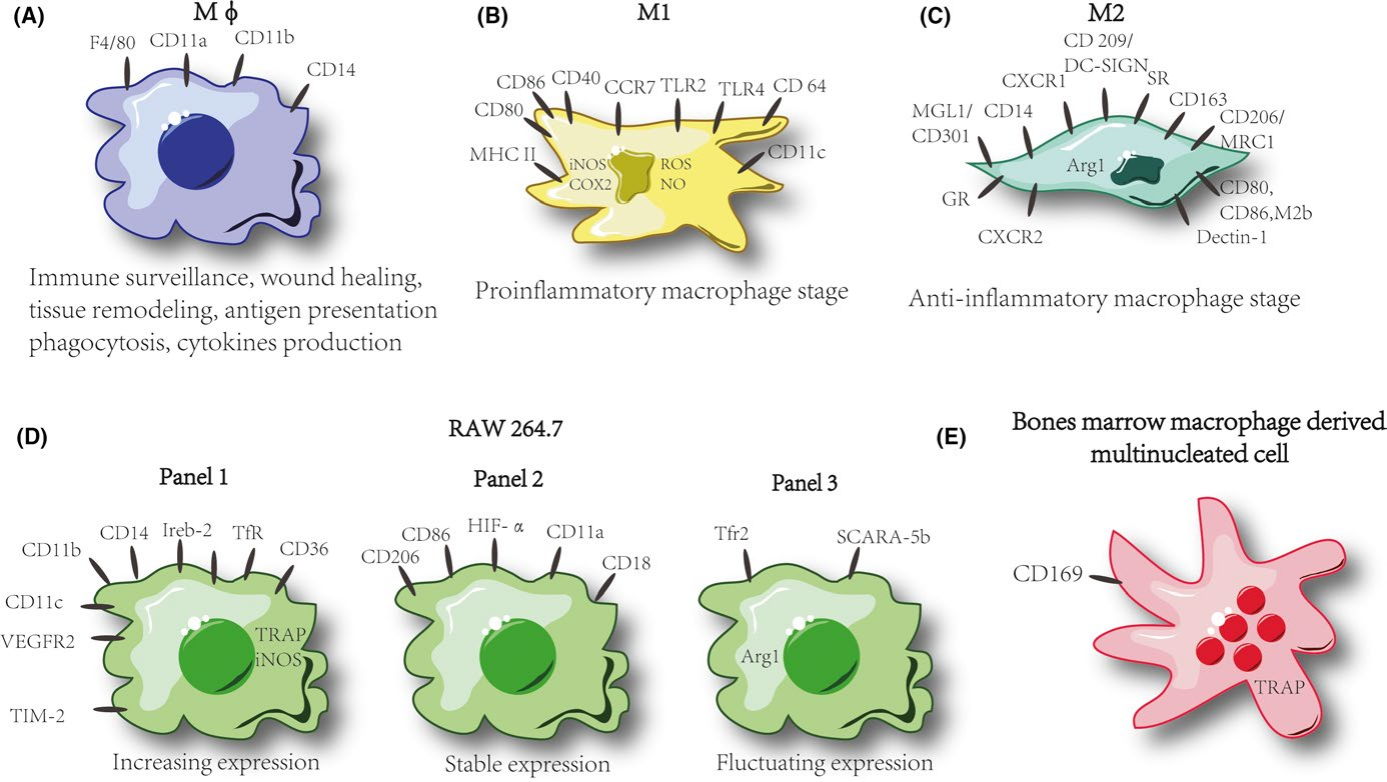
Schematic of macrophage phenotype. (A) Phenotype of M0 macrophage. (B) Phenotype of M1 macrophage. (C) Phenotype of M2 macrophage. (D) Phenotype of three panels of RAW264.7 cell linage. (E) Phenotype of bone marrow macrophages

Similarly, genes in subpanel‐2 and subpanel‐3 also associated with macrophage activation including: CD86, HIF‐1α, CD11a, CD18, CD206, CD200R, Glut1 (Glucose transporter 1) and Ly6c and TfR2, Arg1 and SCARA‐5b (scavenger receptor class A member 5) respectively.^32^ Interestingly, however, as an iNOS inhibitory genes, the Arg1 also highly expressed in another polarized macrophage stage: anti‐inflammatory (M2) phenotype,^49^, ^50^ and its expression in RAW264.7 has be showed significantly higher in the passage of No. 15.^32^ Notably, the expression of TRAP, which involved in osteoclastogenesis and as one of osteoclastic specific genes remains stable in RAW264.7 cell linage from the passage No. 5.^32^ Besides that, Wang et al^51^ have reported that CD109, as a glycosyl‐phosphatidylinositol (GPI)‐anchored protein play a crucial role in RANKL‐induced RAW‐OCs formation and bone resorptive function. The study suggested that CD109 might be a co‐receptor or decoy receptor for TGF‐β that would otherwise bind to cell‐associated TGF‐β receptors, when TGF‐β signalling is down‐regulated by CD109, subsequent inhibition SMAD family member 3 (SMAD3) signalling occurs which might lead to a decrease in the formation of TRAF6 promoted of formation of TAK1 complexes, which comprise the TAK1 catalytic subunit (TAK1), TAK1‐binding protein 1 (TAB1), finally could inhibit RAW‐OCs formation^51^ (Figure 1).

## 3 RANKL AND ITS ROLE IN RAW‐OCS INDUCTION

Receptor activator of nuclear factor kappa B (RANK also known as ODF, OPGL and TRANCE), which is one of TNF receptor family members, is expressed in osteoclasts and their precursor cells as the receptor of RANKL.^3^, ^9^, ^10^, ^52^ Downstream signalling through RANK is essential for osteoclastogenesis.^18^, ^19^ RAW264.7 cell linage has been identified as a transfectable RANK expressing cell linage.^53^, ^54^, ^55^ Hsu et al^56^ firstly reported RANKL induced osteoclastogenic RAW‐OCs. Partly, similar to its counterpart BMM‐OCs, RAW‐OCs derived from the RANKL/RANK binding and NF‐κB activiation, consequently further presented a significant bone resorptive function in vitro.^57^, ^58^ Since then, RAW‐OCs have been extensively employed in osteoclastogenesis studies for more than 20 years.

RANKL‐induced RAW‐OCs have been proved as an important cellular model for bone homeostasis studies because the merits of RAW264.7 cell linage, which lies in its widespread availability, homogeneous nature of primary osteoclasts population (devoid of osteoblasts, lymphocytes and stroma, etc), and ease of culture and transfection for genetic manipulation.^20^, ^27^, ^28^, ^59^, ^60^, ^61^ Otherwise, osteoclast differentiation is dependent upon the intimate cellular interaction of myeloid preosteoclast precursors with either osteoblasts or stromal cells and is influenced by a wide range of local factors.^12^, ^14^, ^62^, ^63^, ^64^ Whereas, RANKL, expressed on both stromal cells and osteoblasts, plays an essential role in the regulation of osteoclast differentiation.^10^, ^65^ Thus, a soluble recombinant form of RANKL (sRANKL) is sufficient to replace fully the requirement for osteoblast and stromal cell interactions in the induction of osteoclast differentiation in in vitro culture system.^66^, ^67^ However, despite the advantages of RANKL for its role in the RAW‐OCs stimulation, there still exist conspiracies during the induction of RANKL‐induced osteoclast from RAW264.7. For instance, l‐glutamine for decades has been recognized as an important factor for cell line growth and differentiation in in vitro culture systems,^70^, ^71^ otherwise the importance of l‐glutamine in osteoclastic cellular culture medium was demonstrated by Indo et al^73^ However, recently Nguyen et al^74^ have reported that during the RANKL stimulated RAW‐OCs induction media, the plus with l‐glutamine at the concentration 4‐6 mM could decrease the total number of multinucleated RAW‐OCs, which could reverse by reducing the concentration of l‐glutamine to 1‐2 mM. Although these results might be because of the accumulation of ammonia/ammonium ion from the breaking down of l‐glutamine in the culture system,^75^ it definitely raised requirements for comprehensive understanding the RANKL for its role in RAW‐OCs induction, which include the efficacy of induction, relevant cellular mechanisms and association of metabolic products.

**Figure 2:**
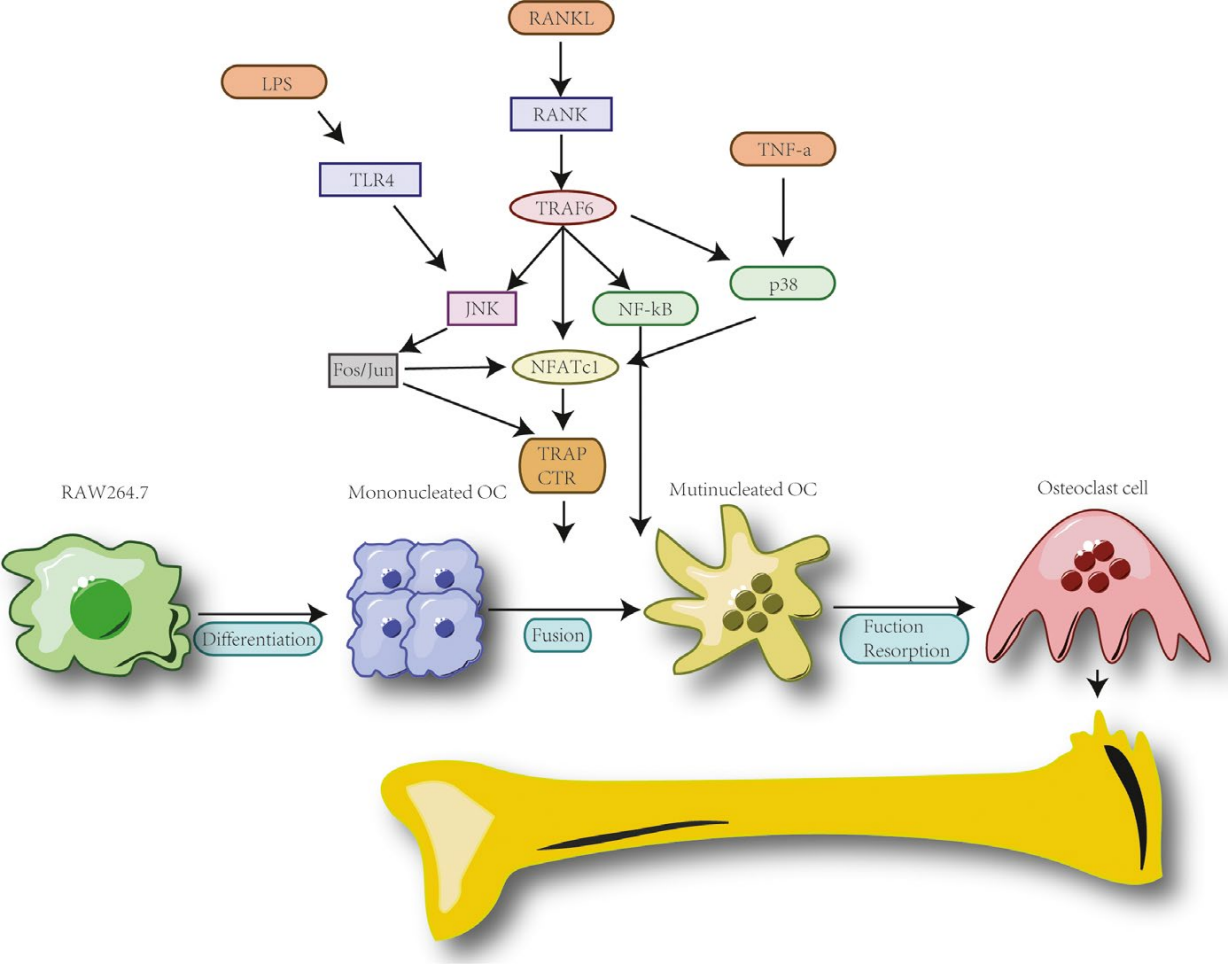
Schematic of RANKL, LPS and TNF‐a signalling for RAW‐OCs

Besides that, Cuetara et al^20^ have reported the osteoclastogenic activity is various among each colony of RAW‐OCs, even in the same passage and same culture condition. Interestingly, however, their study found that all RAW264.7 clones tested expressed the RANKL receptor RANK and expressed the osteoclast marker genes TRAP and cathepsin‐K mRNA with RANKL treatment.^20^ Comparing BMMs, RAW264.7 cell linage are a transformed macrophage like cell line derived from the lymphoma of a male BALB/c mouse infected by the A‐muLV.^76^ The retrovirus encodes an oncogenic form of the *Abelson kinase*, v‐Abl, which is a fusion protein where portions of the retroviral Gag protein substitute regions of the SH3 domain of c‐Abl, rendering the tyrosine kinase constitutively active.^77^, ^78^ Moreover, previous study reported RANKL induced RAW‐OC expressed β3 integrin mRNA,^20^, ^81^ which substitute the regions of SH2 domain.^82^, ^83^ Therefore, the cellular mechanisms of RANKL‐induced RAW‐OCs formation and the bone resorptive activity of RAW‐OC may occur through various signalling cascades cross‐talking, which is the discrepancy in RANKL‐induced BMM‐OCs formation. Specifically, different to BMM‐OCs, document reports have shown that RAW 264.7 cells can proliferate and undergo osteoclastogenic differentiation independently from M‐CSF.^28^, ^85^ The underlying cellular mechanisms, might lie in v‐Abl in RAW 264.7 cells, were limited to M‐CSF‐related signalling pathways. Although these results raise a cautious consideration for using RAW‐OCs in the studying signalling downstream of M‐CSF cascades, it is still acceptable for studying the downstream of RANKL (Figure 2).

RANKL binding with RANK induces the recruitment of tumour necrosis factor receptor‐associated factors (TRAFs) to the cytoplasmic domain of RANK, including TRAF‐2, TRAF‐5 and TRAF‐6.^86^, ^87^, ^88^ This engagement leads to the activation of a signalling cascade with downstream targets, which including extracellular regulated kinase (ERK),^81^ p38 mitogen‐activated protein kinase (p38),^59^ c‐Jun N‐terminal kinase (JNK),^56^, ^89^ phosphatidylinositol‐3 kinase (PI3K)^90^ and IκB kinase.^91^, ^92^ Consequently, crucial osteoclastogenic transcription factors, such as activator protein‐1 (AP‐1:c‐Fos and c‐Jun),^93^ nuclear factor‐κB (NF‐κB),^94^ and nuclear factor of activated T cells c1 (NFATc1) are activated.^27^ In that, NFAT family members are previously been reported expressed in RAW264.7 cells and that their expression is up‐regulated in response to RANKL stimulation.^95^, ^96^ In fact, calcineurin inhibitory peptide could inhibit the RANKL‐induced RAW264.7 monocyte/macrophage cell line into mature RAW‐OCs. Otherwise, ectopic expression of a constitutively active calcineurin‐independent NFATc1 mutant in RAW264.7 cells fused into morphologically giant multinucleated RAW‐OCs, which possess the capabilities for the resorption of a physiological mineralized matrix substrate.^97^, ^98^, ^99^ Meanwhile, as critical transcriptional factors, c‐Fos and NFATc1 are sufficient to induce the expression of osteoclast‐specific genes expression, including TRAP and cathepsin K,^100^ and fusion‐specific genes expression, which including dendritic cell‐specific transmembrane protein (DC‐STAMP).^101^ However, although initial studies speculated that RANKL/RANK signalling is indispensable for RAW‐OCs formation, extensive study explored the understanding of the contingent series of signalling events involved in RAW‐OCs is far from completely understood.

## 4 LIPOPOLYSACCHARIDE AND ITS ROLE IN RAW‐OCS INDUCTION

Physiological defense responding is important for the host to survive infection.^76^, ^102^ Lipopolysaccharide (LPS) as a major component of the outer membrane of Gram‐negative bacterial factor for inflammation has ability to induce the expression of a variety of pro‐inflammatory cytokines and nitric oxide producing enzyme iNOS in macrophage.^30^, ^103^ LPS or the consequent various cytokines elicited in infectious lesions may modulate physiological and pathological osteoclastogenesis respectively. However, further lead to a pathological bone resorptive condition.

RAW264.7 is one of the three cloned cell lines (RAW309 and WR19M), which have been established from murine tumours induced with *Abelson leukemia* virus and express properties of macrophages.^76^ Macrophages (Mφ or M0) are inflammatory cells with high capacity for engulfing and digesting pathogens and cell debris.^104^ Otherwise, based on the micro‐environment, macrophages play increasingly defined roles in orchestrating the healing of various damaged tissues and show high heterogeneity, plasticity and adaptation abilities.^105^, ^106^ Specifically, during the inflammatory osteoclastogenesis, in response to multiple signals or cytokines, macrophages might differentiate into different types of multinucleated cells to internalize the large amounts of un‐necessaries such as strong inflammatory induced host cells, and wear debris, which originally from orthopedic instruments.^104^ During this process, macrophage changing their morphologies by cellular fusion, further formed into multinucleated giant cells (MGCs), which bear the function for engulf and digestion.^104^ In fact, osteoclasts are one of MGC cells, which are attached tightly to the bone surface, and secrete protons and lysosomal enzymes for bone resorption.

Initially, LPS has only been suggested to promote the differentiation and survival of BMM‐OCs through generating kinds cytokines, such as PGE2, IL‐1, RANKL and TNF‐α.^107^, ^108^ Interestingly, although previous studies have demonstrated RANKL and M‐CSF are essential and sufficient for BMM‐OCs induction,^85^, ^109^ recent studies have suggested that M‐CSF might not participate in the LPS‐induced RAW‐OCs in vitro culturing system.^28^, ^30^ This speculation proved by the anti‐M‐CSF antibody could not inhibit LPS‐induced RAW‐OCs formation and M‐CSF could not be detected in the supernatant from cultures of LPS treated RAW264.7 cells, besides pretreatment with M‐CSF has not shown evidence for enhancing the RAW‐OCs formation. Otherwise, RANKL do not seem to be involved in LPS‐induced osteoclast formation from RAW264.7 either. Islam et al^110^ firstly demonstrated that LPS causes the formation of TRAP‐positive MGC in RAW264.7 cells and that they exhibit a pit‐forming activity on calcium carbonate‐coated plates. These results suggested that RAW264.7 cells might act as osteoclast progenitors and could differentiate to osteoclasts in response to LPS. Meanwhile, Hotokezaka et al^111^ have reported LPS‐induced RAW264.7 cells fusion and osteoclastic RAW‐OCs formation by RANKL‐independent manner. However, the molecular mechanisms underlying the LPS‐induced RAW‐OC formation still remain unclear. One speculation is that LPS might mimic RANKL‐induced osteoclast formation via activation of NF‐κB and SAPK/JNK, because the inhibition of SAPK/JNK by the selective inhibitor (SP600125) could inhibit LPS‐induced osteoclastogenesis^112^ (Figure 2).

Interestingly, on the contrary, Nakanishi‐Matsui et al^113^ reported that LPS‐induced multinuclear cells did not express osteoclast‐specific enzymes including TRAP and cathepsin K. The reason for their speculation lying in the period of osteoclast formation is observed at third to seventh day after RANKL induction in RAW264.7. Whereas, the multinucleated cells derived from RAW264.7 cells are formed within 16 hr after LPS induction.^113^ However, this rapid formation should be reasonable because the response to LPS should correspond to that to bacterial infection.^113^ Moreover, LPS‐induced RAW‐OCs formation does not require the assistance of other cells, whereas primary osteoclast precursors require RANKL, which secreted from osteoblasts. Otherwise, although RAW264.7 cell linage has been identified as a transfectable RANK expressing, the LPS‐induced RAW‐OC was associated with RANKL‐independent signalling cascades.^111^ Besides that, even in Nakanishi‐Matsui study, they found these LPS‐induced giant multinucleated cells manifested as a promising internalization ability for polystyrene beads (diameter 6‐15 μm),^113^ this cellular activities similar to the osteoclasts were induced by periprosthetic wear debris. Further, they found the internalizing efficacy of LPS‐induced multinucleated cells is better for osteoclast.

**Figure 3:**
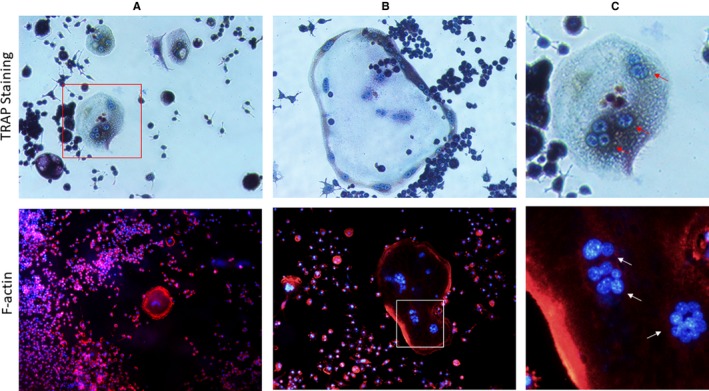
Small and big RAW‐OCs. (A) Small RAW‐OCs with the average 3‐5 nucleus. (B) Big RAW‐OCs with average nuclei number > 10. (C) Box magnified. (Red and white arrows for TRAP staining and F‐actin nuclei respectively)

Studies, including ourselves previous report, stimuli could induce RAW264.7 fuse into giant sized RAW‐OCs differentiate.^28^, ^113^ In fact, depending on the species, osteoclasts involved in normal bone remodelling contain an average of 3‐10 nuclei.^114^ Based on this, conventional osteoclast could be defined as TRAP‐positive stained nucleus number greater than 3. Interestingly, however, according to the number of nucleus, osteoclasts could be classified into ‘big osteoclast’ and ‘small osteoclast’, which containing the nuclei number greater than 10 or among 2‐5 in each osteoclast respectively^115^ (Figure 3). Pathological study demonstrated that the large osteoclast dominated in inflammatory induced bone loss, which leads to extensively bone resorption, such as Paget's disease and periodontal diseases. Piper et al^114^ have demonstrated a decrease in volume of resorption per nucleus as the number of nuclei per cell increased. Moreover, other study demonstrated that approximately 40% of the large osteoclastic multinucleated cells were actively resorbing in the population while only 6% of the small osteoclastic multinucleated cells were doing likewise. Lees et al^116^ have showed the resorptive activity associated with intracellular pH, which presented lower level in the large and functional osteoclasts comparing to small and non‐resorptive osteoclasts, respectively. Otherwise, Manolson et al^117^ showed that ‘α3’ V‐ATPase, as the critical enzyme of osteoclast PH regulator, expression was significantly increased in the larger RAW‐OCs compared to smaller RAW‐OCs.^118^

In fact, LPS presented a dual modulating effect on BMM‐OCs induction. Zou et al^119^ showed LPS has not exhibit osteoclastogenic activity on BMMs, on contrary, their study has shown LPS inhibited RANKL‐induced osteoclast differentiation in the absence of stromal cells or osteoblast. Otherwise, our previous study showed pretreatment with M‐CSF and LPS has not shown any up‐regulatory effects on lower concentration of RANKL‐induced RAW‐OCs.^28^ These results consistently with one previous study results that LPS inhibits the osteoclast formation in whole bone marrow cells with 1,25‐dihydroxyvitamin D3 via GM‐CSF production.^120^ However, previous study speculated that GM‐CSF might play a crucial factor for suppressing the LPS‐induced BMM‐OCs formation.^121^

However, as a critical potential inducer, LPS could enhance the osteoclastogenesis in RANKL‐pretreated BMMs,^122^ even if present in the absence of exogenous RANKL. Document interesting studies reported that LPS enhances osteoclast formation in cultures of whole bone marrow cells with dexamethasone and markedly enhances it in the presence of 1,25‐dihydroxyvitamin D3 and dexamethasone.^123^, ^124^ Otherwise, LPS stimulates osteoclastic bone resorption in vivo and in vitro in organ culture and increased osteoclast differentiation in whole bone marrow cell culture.^30^ Although, molecular study showed LPS does not exhibit osteoclastogenic property in the absence of osteoblasts or stromal cells, LPS could indirectly involved in promoting osteoclastogenesis. In that, LPS enhance RANKL expression in osteoblasts through Toll‐like receptors (TLRs),^125^ meanwhile LPS stimulates various cytokines secreting in micro‐environment, including IL‐1, PGE2 and TNF‐α, which involved in LPS‐mediated bone resorption.^126^ Interestingly, however, on the contrary to their counterpart, LPS‐induced RAW‐OCs formation in different fashion. Despite the aforementioned NF‐κB and SAPK/JNK signalling, LPS could up‐regulate MAPK and COX‐2 in RAW264.7 cells,^30^ which are the downstream signalling of RANKL/RANK. Therefore, LPS regulates the osteoclast formation in a complicated fashion, which presenting discrepancies among BMM‐OCs and RAW‐OCs induction, and associated molecular signalling. Therefore, depending on the experimental design and various cell lineage choice criteria, LPS could manifest an inhibitory effect or presenting induces on osteoclastogenesis.

## 5 TNF‐α AND ITS ROLE IN RAW‐OCS INDUCTION

TNF‐α, which is produced by many types of cells including monocytes and macrophages, has been proved to involve in bone resorption, particularly in inflammatory bone diseases such as rheumatoid arthritis^127^ and aseptic periprosthetic osteolysis.^128^ TNF‐α have been speculated to act directly by enhancing proliferation and activity of cells in the osteoclast lineage, or indirectly by affecting the production of osteoclast differentiation factors via osteoblast/stromal cells such as RANKL and its soluble decoy receptor, osteoprotegerin (OPG).^111^, ^129^

Despite the regulatory effects of TNF‐α on RANKL‐induced BMM‐OCs formation, recent studies focused on examining whether TNF‐α can promote osteoclastogenesis independently of RANKL signalling for both BMMs and RAW264.7 cell linages.^111^ However, different results among various research groups, for instance, has been reported that TNF‐α could promote osteoclast formation in vitro despite the RANKL signalling blockade,^130^, ^131^ while other reports have demonstrated that RANKL priming in osteoclast precursors is necessary for TNF‐α‐induced osteoclastogenesis.^130^ Further, TNF‐α is not necessarily required for osteoclastogenesis, erosive arthritis or osteolysis, as all these events could occur in the absence of TNF‐α. Therefore, these discrepancies reflected complicate molecular fashion during osteoclastogenesis, which involved multiple signalling cascades cross‐talking, cellular genetic backgrounds and various osteoclastogenic differentiate stages or culture conditions.^132^, ^133^ Thus, the relevance between RANKL and direct/indirect TNF‐α action in osteoclastogenesis remains unclear.

Nakao et al^134^ found that RANKL induces TNF‐α mRNA expression and secretion of TNF‐α protein in both spleen‐cell derived osteoclasts precursors (125 pg/mL) and RAW264.7 cells (600 pg/mL). However, this amount of TNF‐α (0.1‐1.0 ng/mL) by itself did not induce osteoclastogenesis in the absence of RANKL. They suggested that TNF‐α serves as an autocrine factor for osteoclastogenesis in cooperation with RANKL.^135^ Specifically, Zou et al^136^ explained this autocrine manner as RANKL induction of osteoclastogenesis is accompanied by a rapid and transient increase in TNF‐α mRNA abundance in the precursor cell; specific anti‐ TNF‐α antibodies or antibodies directed against TNF receptor type I inhibit the osteoclastogenic activity of RANKL in RAW264.7 cells. Therefore, it is reasonable for the speculation that TNF‐α induced RAW‐OC cross‐talking with RANKL signalling cascades. In fact, AU‐rich elements (AREs) of 3′ untranslated region (3′‐UTR), as cis‐element, involved in the regulation of TNF‐α mRNA transcription.^137^, ^138^ Otherwise, 3′‐UTRs associated with p38 MAPK/SAPK2 cascade induced the c‐Fos mRNA expression,^139^, ^140^ which is a crucial transcriptional factor of osteoclastogenesis. Moreover, p38 MAPK signalling pathways is also implicated in mediating RANKL‐induced osteoclastogenesis, suggested the increased TNF‐α mRNA abundance induced by RANKL is mediated by activating this signalling cascade leading to TNF‐α transcriptional stabilities. Besides that, most recently, Shinohara et al^141^ have reported that double‐stranded RNA‐dependent protein kinase (PKR) association is necessary for TNF‐a induced RAW‐OCs formation by p38MAPK, and ERK signalling (Figure 2).

## ETHICS APPROVAL AND CONSENT TO PARTICIPATE

All animal care and experimental procedures were approved by Animal Care Committee of Hong‐Hui Hospital, Xi'an Jiaotong University College of Medicine (Animal Ethics Approval #1002017019) and conducted strictly followed by ‘the institutional guidelines for the care and use of laboratory animals at the Jiaotong University College of Medicine’.

## CONSENT FOR PUBLICATION

The manuscript is approved by all authors for publication.

## AVAILABILITY OF DATA AND MATERIALS

All data and materials were included in the manuscript.

## CONFLICT OF INTEREST

The authors declare that they have no competing interests.

## AUTHORS’ CONTRIBUTIONS

WS, LK: conception and design, analysis and interpretation of data; draft the manuscript and revise it critically for important intellectual content; final approval of the version to be published. LK, DH: acquisition of data, analysis and interpretation of data; LK: conception and design, revise the manuscript critically for important intellectual content, final approval of the version to be published, account for all aspects of the work in ensuring that questions related to the accuracy or integrity of any part of the work are appropriately investigated and resolved. All the authors read and approved the final manuscript.
